# MicroRNA-155 promotes bladder cancer growth by repressing the tumor suppressor DMTF1

**DOI:** 10.18632/oncotarget.3755

**Published:** 2015-04-18

**Authors:** Yang Peng, Wen Dong, Tian-xin Lin, Guang-zheng Zhong, Bei Liao, Bo Wang, Peng Gu, Li Huang, Yun Xie, Fu-ding Lu, Xu Chen, Wei-bin Xie, Wang He, Shao-xu Wu, Jian Huang

**Affiliations:** ^1^ Department of Urology, Sun Yat-sen Memorial Hospital, Sun Yat-sen University, Guangzhou, People's Republic of China; ^2^ Department of Medical Examination Center, Sun Yat-sen Memorial Hospital, Sun Yat-sen University, Guangzhou, People's Republic of China; ^3^ Guangdong Provincial Key Laboratory of Malignant Tumor Epigenetics and Gene Regulation, SunYat-Sen Memorial Hospital, SunYat-Sen University, Guangzhou, People's Republic of China

**Keywords:** microRNA-155, bladder cancer, DMTF1, Arf, cell proliferation

## Abstract

MicroRNA-155 (miR-155) is dysregulated in human cancers. In this study, we reported that miR-155 was over-expressed in bladder cancer tissues. We found that miR-155 promoted cell proliferation *in vitro* and tumorigenesis *in vivo*. MiR-155 directly reduced the expression of the tumor suppressor DMTF1. The expression of DMTF1 was decreased in bladder cancer tissues. Similar to the restoring miR-155 expression, knockdown of DMTF1 promoted cell growth and cell cycle progression, whereas DMTF1 over-expression rescued the effect of miR-155. Moreover, we investigated DMTF1-Arf-p53 pathway and found that DMTF1 worked in both p53-dependent and p53-independent manners. Taken together, our findings suggested that miR-155 functions as a tumor promoter in bladder cancer, which is partially through repressing DMTF1 expression. The identification of miR-155 and its novel target DMTF1 will be valuable in developing diagnostic markers and therapeutic applications for bladder cancer.

## INTRODUCTION

MicroRNAs (miRNAs) are highly conserved small RNA molecules that might regulate events such as aging and cancer progression [1]. In brief, mature miRNAs, which contain 20 ~ 23 nucleotides, are processed from pri-miRNAs (primary long transcripts) with multiple factors involved [2–4]. Generally, miRNAs directly bind to some sequence-specific sites of target genes' 3′UTRs (3′ untranslated regions) [5], which lead to inhibition of these genes expression [6]. Increasing evidences have confirmed that ectopic miRNAs are key regulatory factors in various types of cancers [7–9].

Bladder cancer is one of the most common genitourinary malignancies around the world [10]. In 2013, there were about 72,570 new cases (4.4% in all cancers) of bladder cancer in the USA, with estimated deaths of 15,210 (2.6% in all cancers) [11]. So far, many molecular biomarkers have been investigated in bladder cancer for recurrence prediction, diagnosis, and even prospective therapy [12–14]. It has been reported that many aberrant miRNAs are key regulators for bladder cancer progression. However, the molecular mechanisms are yet clearly elucidated, which makes it necessary to identify novel miRNAs.

In the present study, we demonstrated that miR-155 was over-expressed in bladder cancer tissues. *In vitro* study showed that over-expression of miR-155 promoted cell proliferation, colony formation and cell cycle progression. DMTF1 was confirmed to be a direct target of miR-155. Furthermore, we studied the tumor suppressive pathway DMTF1-Arf-p53 and its working mechanism. Taken together, this report suggested that miR-155 promotes bladder cancer growth by directly repressing the tumor suppressor DMTF1.

## RESULTS

### Over-expression of miR-155 in human bladder cancer tissues

**Figure 1:**
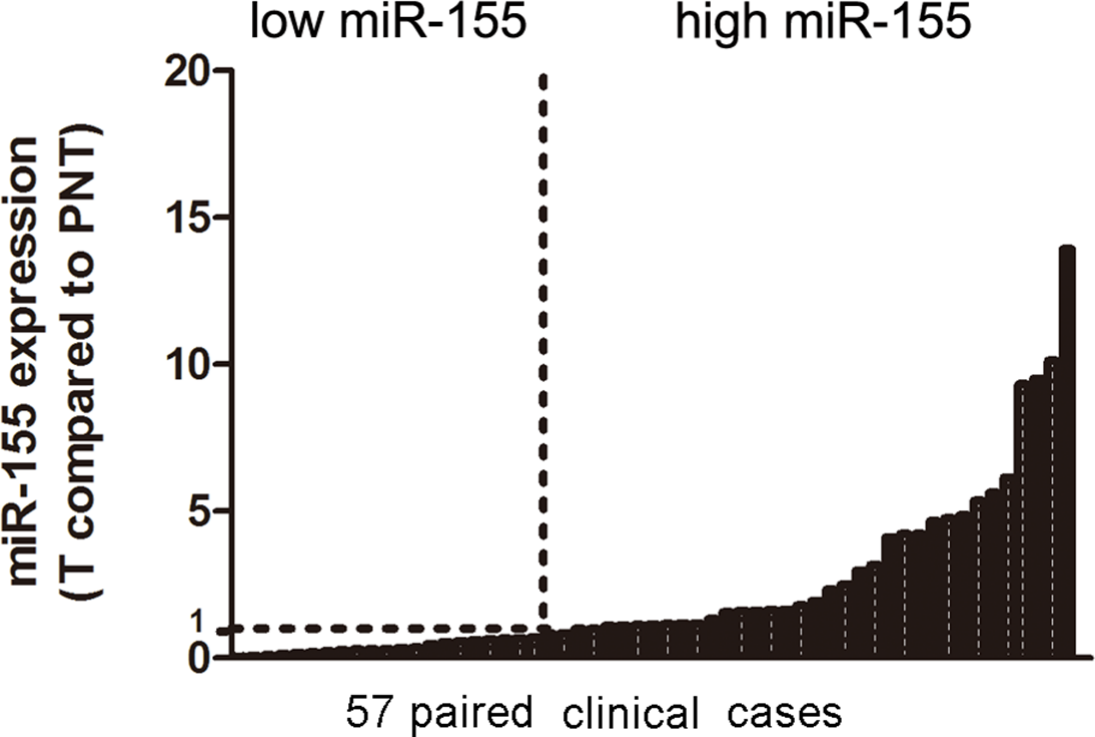
Analysis of miR-155 in clinical tissues Quantitative Real-time PCR of miR-155 was performed in 57 cases of bladder cancer patients. Data were normalized to paired normal tissues (PNT). T: Tumor tissues.

As previously reported [13], microarrays of miRNAs were conducted in 6 pairs of bladder cancer and adjacent normal tissues. Among those highly expressed miRNAs, miR-155 got a moderate score and ranking. However, miR-155 is of interest because: 1) there are no preclinical research about miR-155 in bladder cancer so far; and 2) miR-155's role in different cancers is paradoxical, with oncogenic tendency in breast cancer [15] and renal cancer [16], while anti-tumorigenic potential in gastric cancer [17] and ovarian cancer [18]. Therefore, we further validated miR-155 expression through RT-qPCR in 57 independent pairs of bladder cancer and normal adjacent tissues, where 32 (56.14%) cases showed a higher level of miR-155 (Figure 1). Statistical analysis revealed that miR-155 over-expression associated positively with tumor stage and tumor size (Table 1). There was no significant correlation between miR-155 over-expression and patients' gender, age, tumor grade and recurrence.

**Table 1:** Correlations between miR-155 expression and clinical characteristics

Characteristics	Cases number	Mir-155 expression		*P* value	Chi-square
		high	low		
**Total**	57	32	25		
**Gender**					
Male	49	26	23	2.167	0.141
Female	8	2	6		
**Age**					
<65	31	16	15	0.566	0.452
≥65	26	16	10		
**Tumor stage**					
Ta, Tis, T1	14	4	10	5.728	0.017^*^
T2-T4	43	28	15		
**Lymph node**					
N0	47	27	20	0.186	0.667
N1, N2	10	5	5		
**Tumor grade**					
G1	16	8	8	0.341	0.559
G2, G3	41	24	17		
**Tumor size**					
<3	20	7	13	5.592	0.018^*^
≥3	37	25	12		
**Recurrence**					
+	18	12	6	1.184	0.277
−	39	20	19		

* *P* < 0.05

### MiR-155 promotes cell proliferation *in vitro*

**Figure 2:**
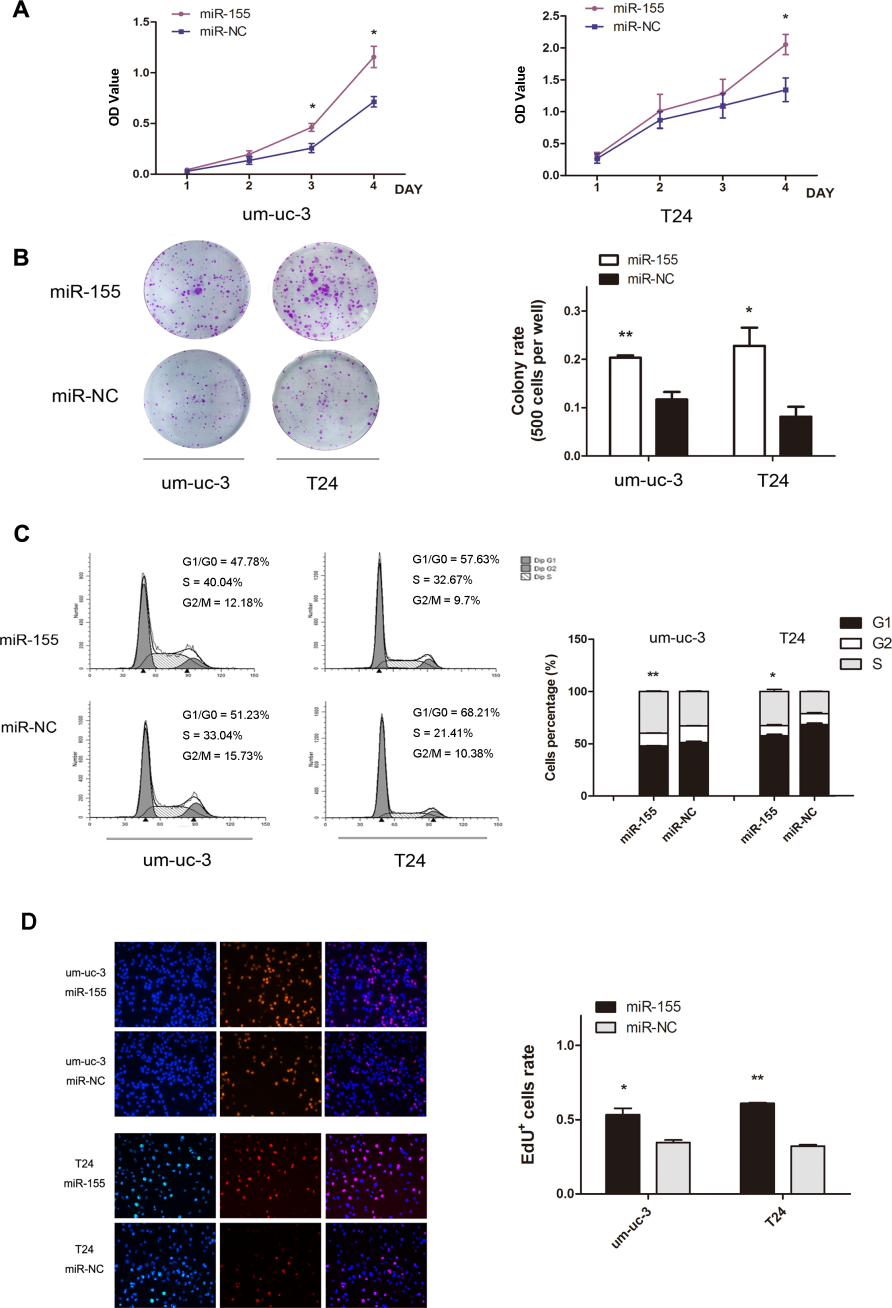
MiR-155 promotes proliferation of bladder cancer cells **A.** MTS assay on um-uc-3 and T24 cells. Absorbance value (OD Value) was used to indicate cell numbers. **B.** Colony formation assay for miR-155-transfected cells (500 cells per well), compared to control NC cells. **C.** Flow cytometry analysis of cell cycle distribution (48 h after transfection) and histograms of cell percentages in each phase. **D.** EdU+ (EdU positively stained) cells were examined under a fluorescent microscope. Blue color represented the nucleus and red color indicated S phase cells. Data shown here were representative images of individual groups (*n* = 3 per group) from three independent experiments. All results were presented as the means ± SD; *n* = 3. (*P < 0.05; **P < 0.01).

To understand the effect of miR-155 on cell proliferation, um-uc-3 and T24 cells were transfected with miR-155 mimics and miR-NC (negative control). MTS and colony-formation assay showed that over-expressed miR-155 increased the ability of cell growth and colony formation (Figure 2A, B; *P* < 0.05). Forty-eight hours after transfection, flow cytometry analysis showed that miR-155 groups had a significant increase of cell proportions in S phase, and a decrease in G1 phase, than that in control groups (Figure 2C; *P* < 0.05). These results were strengthened by EdU assay, which was a more sensitive way to analyze cells in S phase. The number of EdU positively stained cells was significantly higher in miR-155 groups (Figure 2D; *P* < 0.05).

**Figure 3:**
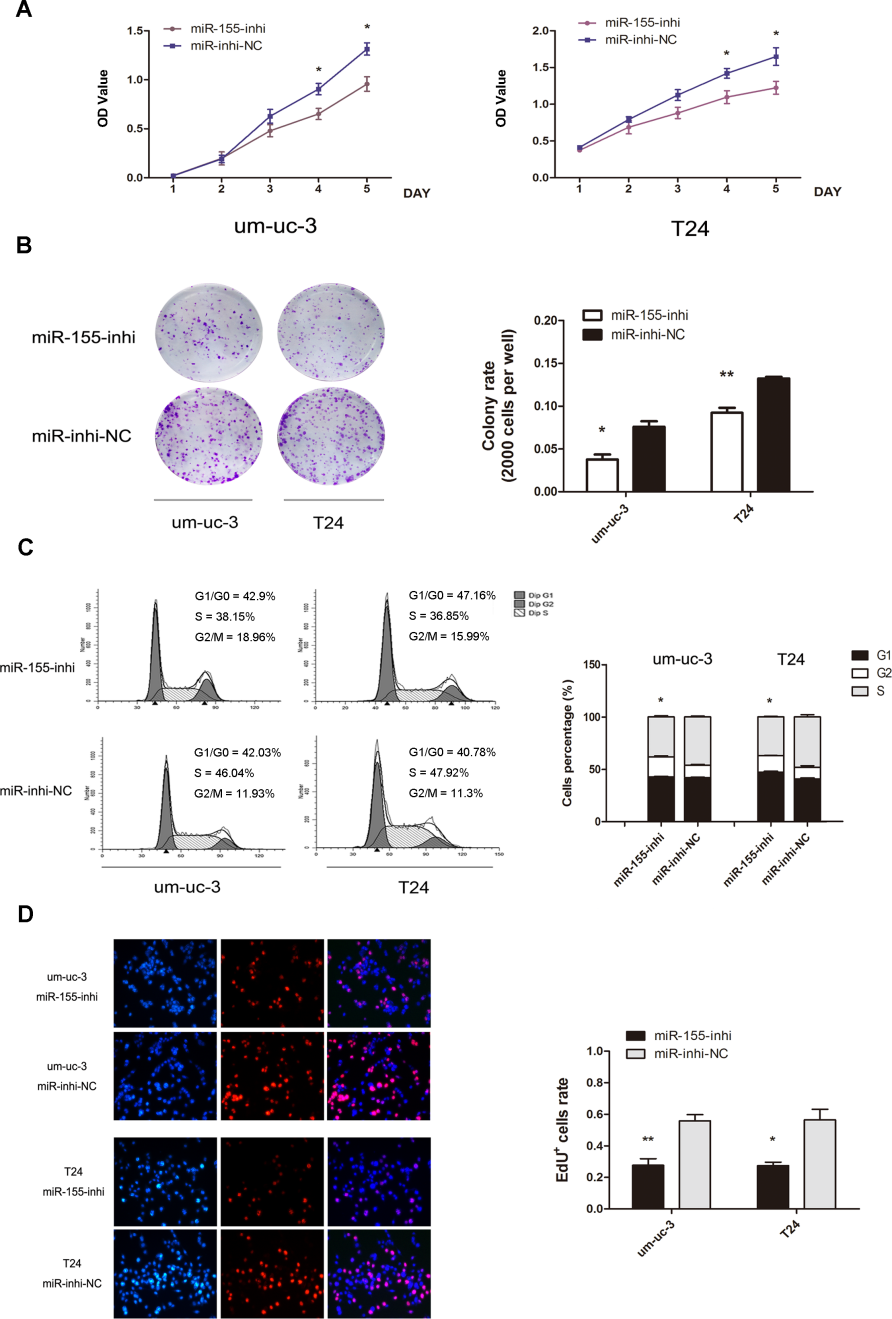
Inhibition of miR-155 decreases cell growth of bladder cancer cells **A.** MTS assays were performed on um-uc-3 and T24 cells with miR-155 inhibitor or inhibitor-NC transfected. **B.** Colony formation (2000 cells per well) and statistical analysis. **C.** Cell cycle analysis of miR-155 inhibition showed decreased proportions of cells in S phase. **D.** EdU assay on cell with transfection of miR-155-inhibitor and statistical analysis. EdU data were representative images of individual groups (*n* = 3 per group) from three independent experiments. All results were presented as the means ± SD; *n* = 3. (*P < 0.05; **P < 0.01).

Loss-of-function experiments were performed, by transfecting miR-155 inhibitor and inhibitor-NC. In contrast to above results, miR-155 inhibitor groups showed decreased cell proliferation and colony formation (Figure 3A, B; *P* < 0.05). Likewise, inhibition groups demonstrated fewer cells in S phase, with more cells in G1 phase (Figure 3C; *P* < 0.05). Fewer EdU stained cells were detected in miR-155 inhibition groups (Figure 3D; *P* < 0.05). These results suggested that miR-155 promotes proliferation of bladder cancer cells.

### DMTF1 is a direct target of miR-155

**Figure 4:**
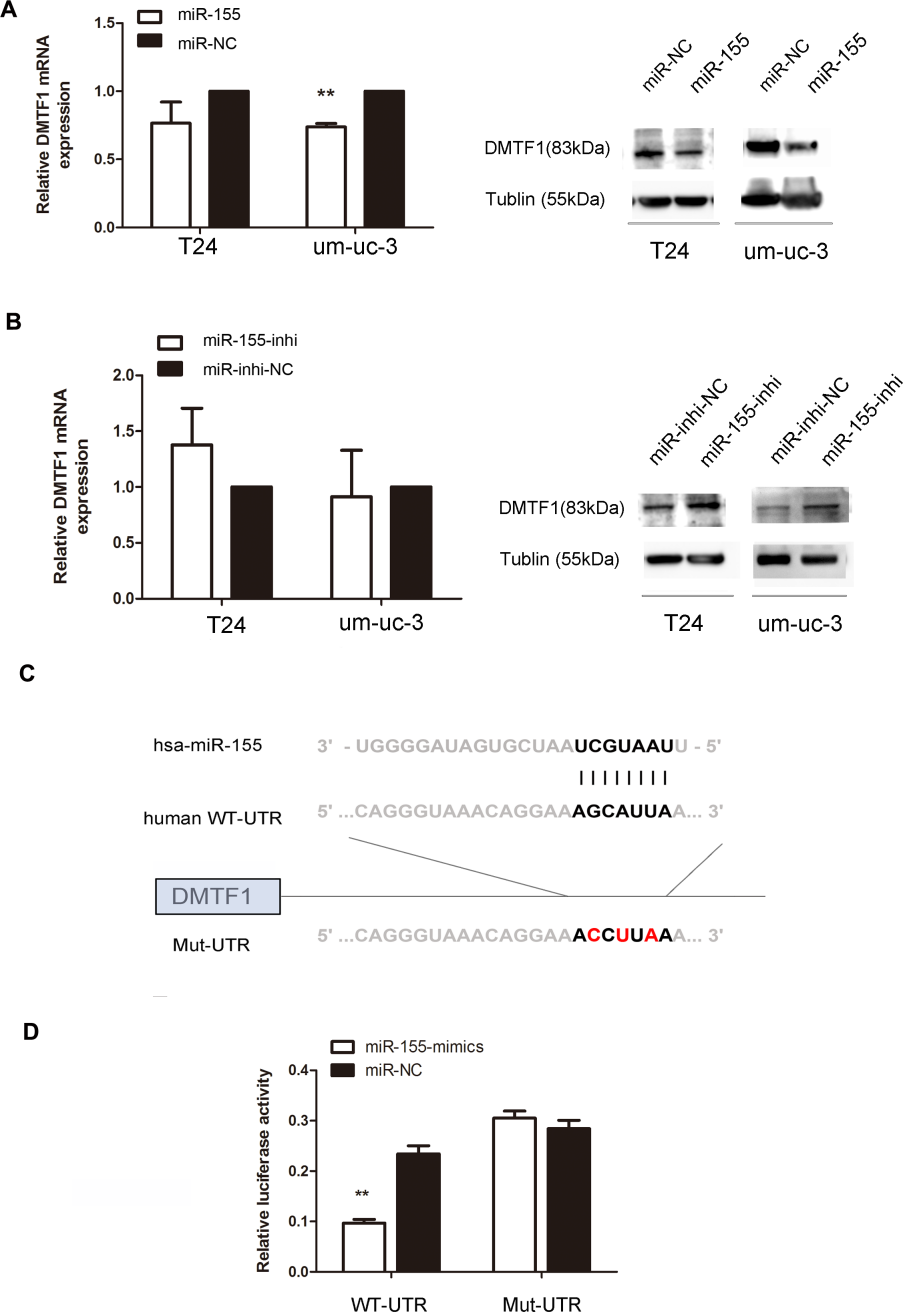
DMTF1 is a direct target of miR-155 in bladder cancer cells **A–B.** Endogenous DMTF1 protein and mRNA levels after miR-155 over-expression or miR-155 inhibition. **C.** miR-155 sequences and its predicted binding site in 3′UTR of DMTF1, with vectors containing wild-type or mutant sequences. **D.** Luciferase assay in H293T cells, with co-transfection of WT or Mut 3′UTR and miR-155 as indicated. Luciferase activity ratios were presented as *firefly luciferase vlaues*/*renilla luciferase values*. RT-qPCR analysis was performed 24 h after transfection. Western blotting analysis was conducted 48 h after transfection. All experiments were performed in triplicate. Results were shown as the means ± SD. (*P < 0.05; **P < 0.01).

We searched the TargetScan (http://www.targetscan.org) to identify target genes, especially those related to cell growth. Among all predicted targets of miR-155, DMTF1 caught our attentions for its tumor suppressive role to induce cell cycle arrest. Therefore, we investigated the effect of miR-155 on DMTF1 mRNA and protein expressions, respectively. We found that mRNA expression of DMTF1 was decreased by miR-155-mimics in um-uc-3 cells (Figure 4A; *P* < 0.01). However, DMTF1 mRNA levels were similar in T24 cells, with transfection of either miR-155 or miR-155 inhibitor (Figure 4A, B; *P* > 0.05). Then we found decreased levels of DMTF1 protein in miR-155 mimics groups of both um-uc-3 and T24. Inversely, the inhibitor groups presented higher expressions of DMTF1 when compared to that in control groups (Figure 4A, B).

To assess whether miR-155 directly binds to 3′UTR of DMTF1, we performed luciferase assay. The target sequence of DMTF1 3′UTR (WT-UTR) or the mutant sequence (Mut-UTR) was cloned into luciferase reporter vectors. H293T cells were transfected with WT-UTR vector or Mut-UTR vector and miR-155 mimics (miR-NC as control) (Figure 4C). The results showed that miR-155 caused a significant decrease of luciferase value in WT-UTR groups compared to that in NC groups, whereas Mut-UTR showed no significant response to miR-155 (Figure 4D; *P* < 0.01). Taken together, it was indicated that DMTF1 is a target of miR-155.

### DMTF1 counteracts miR-155's oncogenic effect on cell proliferation and cell cycle

**Figure 5:**
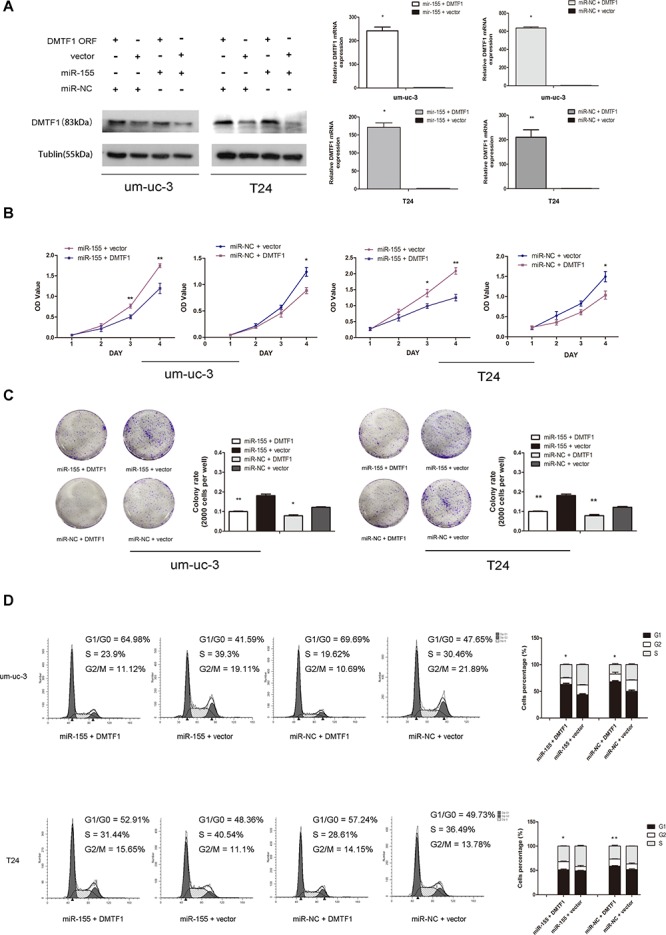
Rescue experiment to confirm DMTF1 as a target of miR-155 **A.** Western blotting and qPCR showed reversed effect of DMTF overexpression to miR-155. Co-transfection efficiencies were also confirmed. **B–C.** MTS assay and colony formation analysis to show the effect of DMTF1, compare to that of miR-155 *in vitro*. **D.** Flow cytometry analysis was performed 48 h after co-transfection of DMTF1-ORF (vector as control) and miR-155 (miR-NC as control). All experiments were performed in triplicate. Results were shown as the means ± SD. (*P < 0.05; **P < 0.01).

To further confirm whether DMTF1 is directly suppressed by miR-155, rescue experiment was performed. We cloned the ORF (Open Reading Frame) region of DMTF1 exogenously into vectors. Then we conducted co-transfection of DMTF1-ORF-vector and miR-155 mimics, with none-vector and miR-NC oligos as controls, respectively. Transfection efficiency was confirmed by RT-qPCR (Figure 5A; *P* < 0.05). Consistent with previous results, *miR-155 + vector* groups showed suppressed DMTF1 protein levels than *miR-NC + vector* groups. DMTF1-ORF greatly reversed miR-155's inhibition on endogenous DMTF1, although *miR-155 + DMTF1* groups and *miR-NC+DMTF1* groups showed no significant difference (Figure 5A). In addition, we tested whether DMTF1 inhibited cell proliferation. MTS and colony formation assay showed that miR-155's oncogenic role was significantly reversed by DMTF1 (Figure 5B, C; *P* < 0.05). Cell cycle analysis further demonstrated that DMTF1 inhibited miR-155's effect on cell cycle progression (Figure 5D; *P* < 0.05). These results further indicated that miR-155 directly represses DMTF1 in bladder cancer cells.

### MiR-155 promotes tumorigenesis potential of bladder cancer *in vivo*

**Figure 6:**
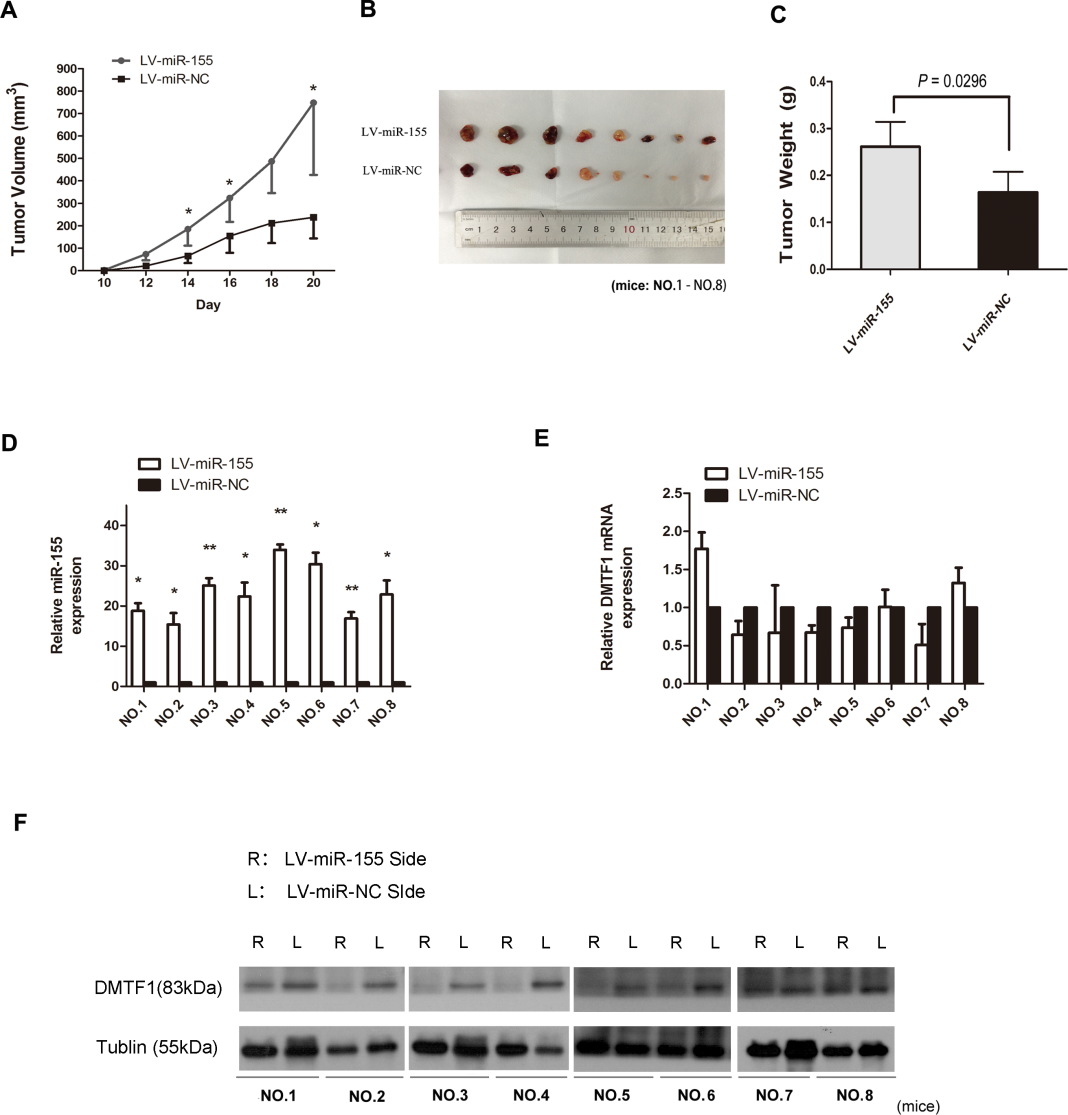
MiR-155 promotes tumorigenesis of bladder cancer *in vivo* **A.** In every two days observation, mean tumor volume from each side was compared. **B–C.** The mean weight of tumors from uc-3-LV-miR-155 injected groups was significantly increased compared to control groups (*P* = 0.0296). **D–E.** In each single nude mouse, miR-155 and DMTF1 mRNA levels were compared between two sides. **F.** Within the same mouse, proteins of tumor tissues from each side were compared, 6 mice showed decreased DMTF1 protein levels in the LV-miR-155 side. L: left side. R: right side. Results were expressed as the means ± SD of 8 mice; *n* = 3. (*P < 0.05; **P < 0.01).

We tested the effect of miR-155 *in vivo*. Lenti-virus containing either miR-155 or miR-NC was used to infect um-uc-3 cells for stable gene expression. Um-uc-3 cells were chosen because of their stronger tumorigenicity i*n vivo* over other cell lines (ATCC; http://www.atcc.org). Two stable cell lines were established: uc-3-LV-miR-155 and uc-3-LV-miR-NC. These cells were then subcutaneously injected into each infrascapular region of nude mouse. As Figure 6A demonstrated, LV-miR-155 sides exhibited significantly increased tumor growth. On day 20, the average volume of tumors from LV-miR-155 sides was bigger than that from the control sides (Figure 6B; *P* < 0.05). The mean tumor weight from LV-miR-155 groups (0.261 ± 0.168 g) was heavier than that from the control sides (0.164 ± 0.139 g) (Figure 6C; *P* < 0.05). Analysis of RNA from tumor tissues showed that miR-155 expression was higher in the LV-miR-155 sides (Figure 6D; *P* < 0.05). But mRNA levels of DMTF1 showed no significant differences between two sides (Figure 6E; *P* > 0.05). However, paired comparison showed that 6 out of 8 mice had a decrease of DMTF1 protein levels in LV-miR-155 sides (Figure 6F). So it indicated that DMTF1 might be inhibited by miR-155 *in vivo*. Taken together, these results suggested that miR-155 promotes tumorigenesis of bladder cancer *in vivo*.

### Knockdown of DMTF1 promotes cell proliferation and cell cycle progression

**Figure 7:**
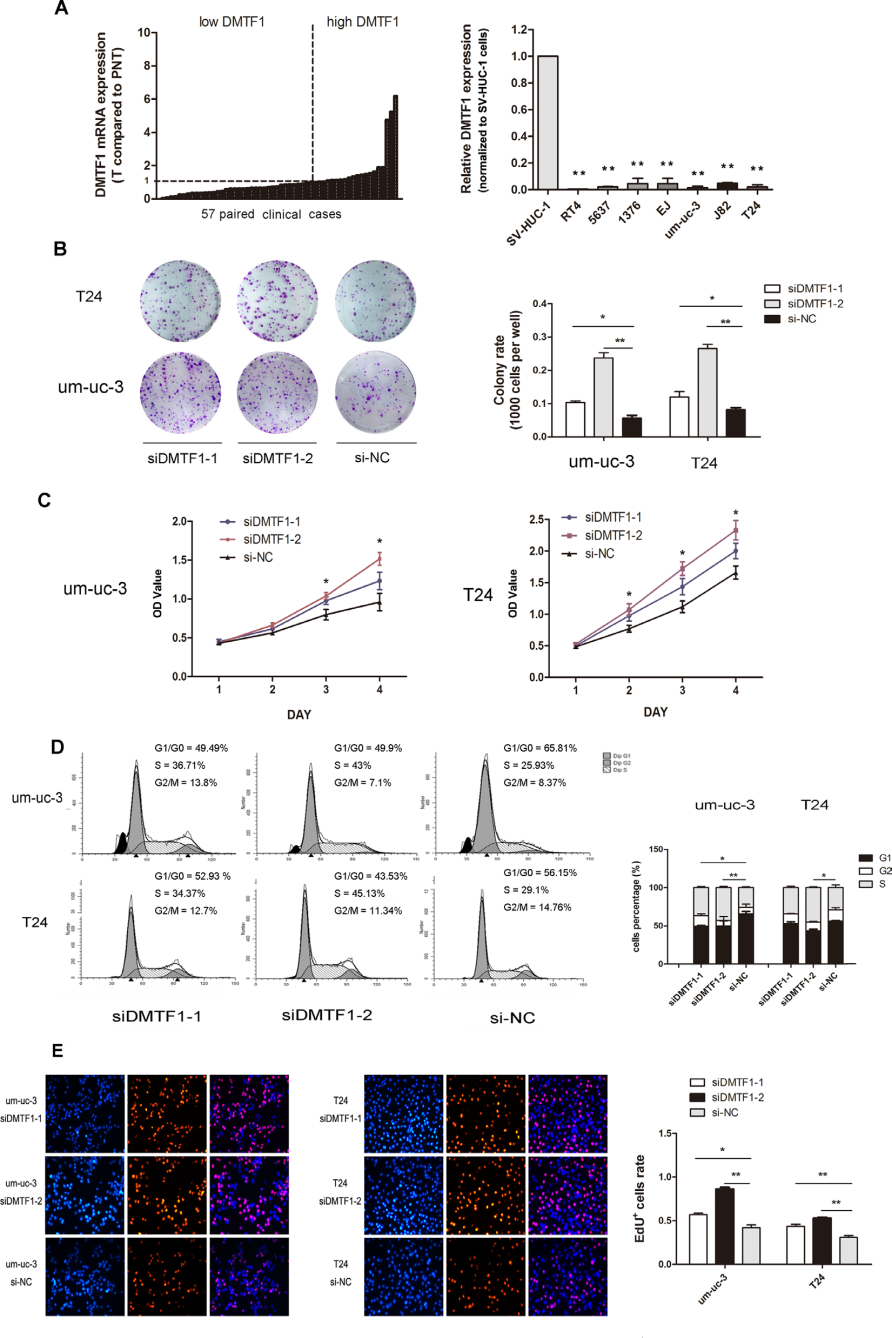
Knockdown of DMTF1 promotes cell proliferation *in vitro* **A.** DMTF1 expression in 57 paired bladder cancer tissues (PNT: paired normal tissues). DMTF1 mRNA levels were detected in 7 bladder cancer cell lines (RT4, 5636, HT1376, EJ, um-uc-3, T24), normalized to SV-HUC-1. **B–C.** Colony formation and MTS assay to show the effect of siDMTF1 on bladder cancer cells. **D.** Cell cycle distribution measured by flow cytometry, with histogram comparison. **E.** Knockdown of DMTF1 significantly increased EdU+ cells compared to that in NC groups. EdU assay showed representative images from individual group (*n* = 3 per group) of three independent experiments. All results were expressed as the means ± SD; *n* = 3. (*P < 0.05; **P < 0.01).

Firstly, we examined the mRNA expression of DMTF1 in those 57 clinical cases mentioned above. As shown in Figure 7A, 35 (61.40%) cases had lower DMTF1 levels when normalized to paired adjacent tissues. In addition, we observed lower mRNA expression of DMTF1 in bladder cancer cell lines when compared to that in the normal uroepithelium cell line SV-HUC-1 (Figure 7A; *P* < 0.01), although miR-155 expression in these cell lines varied (Supplementary Figure S1A).

Then we investigated DMTF1's effect *in vitro*. SiRNAs (si-DMTF1s) were transfected into um-uc-3 and T24 cells. Transfection efficiencies were confirmed through RT-qPCR and Western blotting (Supplementary Figure S1B). In MTS and colony formation assay, si-DMTF1 groups demonstrated significant increase in cell growth and colony formation ability (Figure 7B, C; *P* < 0.05). Cell cycle analysis showed that si-DMTF1 groups had higher proportions of cells in S phase, but fewer cells in G1 phase were detected (Figure 7D; *P* < 0.05). EdU incorporation assay revealed that si-DMTF1 groups had more EdU^+^ cells than control groups (Figure 7E; *P* < 0.05). Taken together with rescue experiment, it was confirmed that DMTF1 suppresses proliferation of bladder cancer cells.

### DMTF1 activates Arf expression to exert anti-tumor effect in both p53-dependent and p53-independent manners

**Figure 8:**
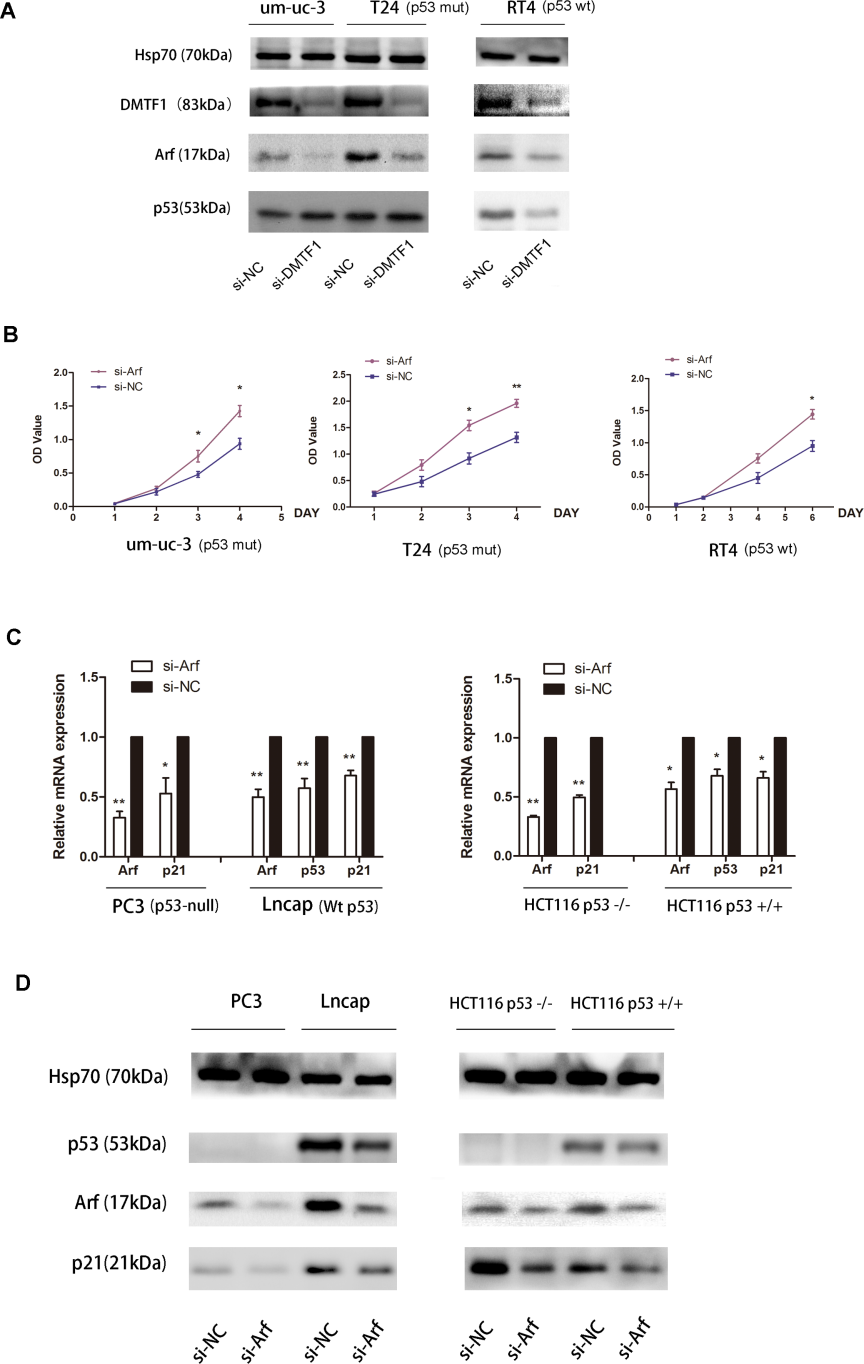
DMTF1-Arf-p53 pathway and functional study **A.** After DMTF1 inhibition, Arf, p53 protein levels were detected by Western blotting. P53-mutant cells: um-uc-3 and T24; wild-type p53 cells: RT4. **B.** MTS assay indicated Arf's effect on cell proliferation, in both mut-p53 (um-uc-3, T24) and wt-p53 (RT4) cells. **C–D.** When Arf was knocked down, p53 and p21 expression in both p53-null cells and p53-wild-type cells were examined by qPCR and Western blotting. Here in Western blotting, Hsp70 (70 kDa) was used as loading control protein instead of Tublin, because molecular weight of Tublin (55 kDa) is close to p53 (53 kDa). All results were presented as the means ± SD; *n* = 3. (*P < 0.05; **P < 0.01).

Previously, DMTF1 was considered as a tumor suppressor by inducing cell cycle arrest in lung cancer and breast cancer [19, 20]. Through directly binding to Arf promoter and activating Arf expression, DMTF1 stimulated the tumor suppressive Arf-p53 pathway. In addition, activated p53 directly induced the expression of the downstream target p21 [21]. So we transfected si-DMTF1 into um-uc-3 and T24 cells, and then examined Arf expression. Transfection efficiency was also confirmed by qPCR (Supplementary Figure S1C). Both cell lines showed decreased protein expressions of Arf when DMTF1 were knocked down. However, there was no significant change of p53 expression (Figure 8A).

Surprisingly, p53 mutation (ATCC) was found in the above two cell lines. Thus, we choose RT4, which is a wild-type p53 bladder cancer cell line, as control. The result showed that knockdown of DMTF1 caused Arf inhibition and slight p53 decrease. In addition, MTS assay showed that si-Arf groups in um-uc-3, T24 and RT4 cells demonstrated an increase in cell proliferation than that in the control groups (Figure 8A, B; *P* < 0.05). So Arf might inhibit cell growth in these bladder cancer cells.

However, it was reported that p53 mutation might silence its tumor suppressive function [22]. In order to further illustrate whether DMTF1/Arf works in a p53-independent manner, we chose more p53-null cell lines to study DMTF1-Arf-p53 pathway. So we turned to (1) prostate cancer: lncap (wild-type p53) and PC3 (p53 deletion); and (2) colorectal cancer: HCT116 p53+/+ and HCT116 p53−/−. These are typical pairs of isogenic human cell lines with or without expression of wild-type p53 [23]. The results showed that si-Arf groups had decreased p21 expression, either with or without wild-type p53. Meanwhile, p53 expression showed slight inhibition by si-Arf in lncap and HCT116 p53+/+ (Figure 8C, D; *P* < 0.05). Taken together, it was indicated that DMTF1 might activate Arf in bladder cancer and that the tumor suppressor Arf works in both p53-dependent and p53-independent fashions.

## DISCUSSION

MiR-155 is one of the earliest and most studied functional miRNAs [24]. Constitutively high levels of miR-155 lead to genomic instability [25] and sustained proliferation of malignant cells [26], not only in leukemia [27], but also in solid tumors, such as hepatocarcinoma [28], non-small cell lung cancer [29], and breast cancer [30]. However, there were few studies about miR-155 in bladder cancers. *Wang G et al* had detected miR-155 expression in urine sediment and supernatant, but not tissue samples [31]. Generally, miR-155 is considered as an onco-miR [32] in different kinds of solid tumors [15]. However, some evidence had demonstrated the tumor suppressive role of this classic onco-miR in different types of cancers [17, 18]. Therefore, it is necessary to investigate miR-155 in bladder cancer.

In this study, we found that miR-155 is an onco-miR in bladder cancer. It was reported that miR-155 increases cell growth and colonigenecity. Flow cytometry analysis indicated that miR-155 promotes cell cycle progression. Nude mice study demonstrated that miR-155 promotes tumorigenesis *in vivo*. Luciferase assay and rescue experiment had further confirmed that DMTF1 is a direct target of miR-155. However, it should be noted that there were many other targets of miR-155, such as FOXO3a, LKB1, TP53INP1, *etc* in cancers. [33, 34]. Therefore, miR-155 might suppress multiple downstream genes, besides DMTF1.

DMTF1, also known as cyclin D-binding myb-like protein-1 (Dmp1), is a well accepted tumor suppressor [35, 36]. By directly activating Arf promoter, DMTF1 shows its tumor suppressive capability of inducing cell cycle arrest via Arf-p53 pathway [20, 37]. So DMTF1 might greatly stimulate the tumor suppressor p53 [38]. In present report, the tumor suppressive role of DMTF1 was studied and confirmed in bladder cancer. In addition, clinical data showed that DMTF1 mRNA expression is significantly lower in bladder cancer tissues.

In lung cancer, DMTF1 is considered as a pivotal tumor suppressor. Abnormality of p53 is a common event in human lung cancers [39]. The activity of p53 is activated by Arf, which functions as a stabilizer of p53 by blocking MDM2, a protein responsible for the degradation of p53 [40]. Loss of heterozygosity (LOH, which also indicates the absence of a functional tumor suppressor gene) of the DMTF1 gene is detectable in approximately 35% of human lung carcinomas, which is found in mutually exclusive fashion with LOH of Arf or that of p53 [41]. Thus, in bladder cancer, we suppose that DMTF1 could activate Arf-p53 pathway. However, how it works remains unknown, because structural and functional p53 defects are also found in over one-half of human urothelial carcinomas [42]. In this report, both um-uc-3 and T24 cells have p53 mutations, but cell proliferation is still repressed by DMTF1. Then we found that DMTF1 activates Arf in both wild-type p53 and mutant p53 bladder cancer cells. In addition, knockdown of Arf also promotes proliferation of these cells (Figure 8B), which was consistent with the effect of knockdown of DMTF1. Importantly, here we reported that Arf works in both p53-dependent and p53-independent manners to activate p21 gene. It is well accepted that Arf's anti-tumor activity is through activating p53 transcription, but several researches indicate that Arf also exerts p53-independent tumor-suppressive function [40, 43, 44]. Although some researchers have argued that p53-independent suppressive effect is less pronounced than in p53-dependent manner [45], there are increasing evidence supporting Arf as an independent tumor suppressor in the absence of p53 [46–48]. Arf has been reported to physically bind to more than 25 other proteins [40, 46]. Some of these proteins, which are involved in ribosome biogenesis, DNA-damage response and apoptosis, have been postulated to be responsible for Arf's p53-independent anti-tumor effect [40]. Therefore, in the situation of bladder cancer with existing p53 mutation, it was indicated that the p53-independent way of Arf's effect might account for DMTF1's role as a tumor suppressor.

Currently, bladder cancer causes approximately 150,000 deaths per year worldwide [49]. Many bladder cancer patients are diagnosed at an advanced stage. The potential role of miRNAs in cancer diagnosis is largely accentuated. For example, it was reported that increased miR-127, miR-34b/c, and miR-9 were associated with lymph node metastasis [50]. In the present study, we detected a higher expression of miR-155 in 57 cases of bladder cancer patients. MiR-155 overexpression is also associated with tumor stage and size in these patients, suggesting that miR-155 may serve as a molecular biomarker for cancer prognosis. However, due to the limitation of case number and tissue collection year (2010–2014), we could not perform Kaplan–Meier analysis, metastasis-free survival and overall survival evaluation.

In summary, our results revealed that miR-155 works as an oncogene in bladder cancer and promotes cell growth and proliferation both *in vivo* and *in vitro*. DMTF1 was confirmed to be directly repressed by miR-155. The tumor suppressor DMTF1 activates Arf to induce cell cycle arrest and inhibit cell proliferation, partially in a p53-independent manner in bladder cancer. However, additional studies are necessary to further understand the underlying mechanism, which will help to develop early diagnostic and therapeutic approaches for bladder cancer patients.

## MATERIALS AND METHODS

### Patient samples

Bladder cancer tissues, together with adjacent normal tissues (at least 3 cm away from the tumor), were obtained from surgical resection in Sun Yat-sen Memorial Hospital from 2010–2014. Soaked into RNA*later*^®^ Solution (AM7021, Ambion^®^), all samples were snap-frozen with liquid nitrogen and stored at −80°C until processed (for total RNA extraction). All tissue samples, with pathological confirmations by two distinctive pathologists in the hospital, were under written consent from patients, and approved for study by the Ethic Review Committees of the hospital.

### Cell culture

Human bladder cancer cells T24, um-uc-3 used in the study were maintained according to the instructions of American Type Culture Collections (ATCC, Manassas, VA). Specifically, T24 cells were cultured in RPMI 1640 (Gibco), and um-uc-3 cells were maintained in Dulbecco's Modifed Eagle's Medium (DMEM). These media were supplemented with 10% fetal bovine serum (Bioind, Israel) and 1% penicillin/streptomycin (Hyclone). Cells were grown in a humidified atmosphere with 5% CO_2_ at 37°C.

### Transfection

MiR-155 mimics, miR-NC, siDMTFs, siArf, as well as siRNA-NC oligos were synthesized by GenePharma (Shanghai). MiR-155 inhibitor and miR-inhibitor-NC were purchased from Ribo Bio (Guangzhou). Both miRNA oligonucleotides and siRNAs were transfected at a working concentration of 50nmol/L with RNAiMAX reagent (Invitrogen).

For stable miR-155 over-expression, we purchased Lenti-virus with miR-155-overexpressed plasmid constructed (LV-miR-155) from GenePharma. According to the working manuals, we infected bladder cancer cells for 24 h and added 0.1% puromycin into medium 48 h after transfection. Cells were analyzed by fluorescence microscopy 48 h after transfection. All oligo sequences, transfection efficiency, and fluorescent screening were in Supplementary Table S1 and Supplementary Figures S1, 2.

### RNA isolation and quantitative real-time reverse transcription PCR

Total RNAs from clinical tissues, nude mice tissues or cell lines were extracted by using RNAiso plus reagent (TaKaRa) according to the working instructions. The quantification of miR-155 was conducted by using SYBR^®^ PrimeScript™ miRNA RT-PCR Kit (TAKARA) for both reverse transcription and PCR. cDNA of DMTF1 was synthesized with the PrimeScript™ RT Master Mix (TaKaRa) and PCR was conducted with SYBR Green PCR Kit (TaKaRa). MiR-155 was normalized to U6 small nuclear RNA (U6-snRNA). DMTF1 mRNA was normalized to GAPDH. Quantifications were measured by a Bio-Rad PCR instrument, using the method of 2^−ΔΔ^CT. All primers were shown in Supplementary Table S1.

### MTS and colony formation assays

For MTS analysis, cells were seeded into 96-well plates at 1000 or 2000 cells/well in a final volume of 200 ul and incubated overnight. 3 hours after 20 ul of MTS (Promega) were added into each well, the cell viability was determined by OD value at the wavelength of 490 mm through SpectraMax M5 reader (Molecular Devices).

For colony formation assay, cells were seeded into 6-well plates at a concentration of 500 or 2000 cells/well, with medium changed every 5 days. After 7–14 days incubation, cells were washed with PBS, fixed with 4% paraformaldehyde and then stained with 0.5% crystal violet. Colony-forming rates were then calculated.

### Cell cycle analysis

A total number of 5 × 10^5^ cells were collected after culturing for 48 h. Then they were washed and re-suspended with PBS, added with frozen 70% ethanol at 4°C for overnight fixation. Then fixed cells were treated with DNA-staining solution (3.4 mmol/L Tris-Cl (pH 7.4), PI, 0.1% Triton X-100 buffer, and 100 mg/mL RNase A). Analysis of cell percentage in each phase of cell cycle was accomplished by fluorescence-activated cell sorting (FACS) flow-cytometry.

### EdU labeling

EdU is a thymidine analog whose incorporation can be used like BrdU to label cells undergoing DNA replication [51]. Cells at a concentration of 5 × 10^4^ cells per well were cultured in 24-well plates for 24 h, and then exposed to 50 μM of EdU for additional 3 h at 37°C. Thereafter, 4% formaldehyde was used for cell fixation for 15 min. Then cells were treated with 0.5% Triton X-100 for 10 min at room temperature. After washed with PBS, cells were treated with 200 μl of 1xApollo^®^ reaction cocktail for 30 min. Subsequently, cells of each well were stained with 200 ul of Hoechst 33342 (5 μg/ml) for 30 min and visualized under a fluorescent microscope.

### Western blotting

Cells or mice tissues were lysed using RIPA buffer (Thermo Pierce) containing protease inhibitors cocktail (Roche) according to the manufacturer's instruction. Proteins lysates were separated by electrophoresis in 10% SDS polyacrylamide gels and transferred to PVDF membranes (Millipore). 1.5 h after blocking in 5% nonfat milk, membranes were probed by primary antibodies that were specific to DMTF1 (1:1000, Abcam), p53 (1:2000, Abcam), Arf (1:2000, Abcam), p21 (1:1000, Cell signaling technology), as well as α-tulin (1:1000, Cell signaling technology) and Hsp70 (1:1000, Cell signaling technology) as reference controls. Horseradish peroxidase-conjugated secondary antibodies (1:1000 or 1:2000, Cell signaling technology) and an Immobilon™ Western chemiluminescent ECL kit (Millipore) were used to detect bound antibody.

### Vector construction and luciferase activity assay

The ORF sequence of DMTF1 was amplified with PCR (TaKaRa) and cloned into pCDNA3.1 (−) vector (Promega). The 3′UTR sequence of DMTF1 containing miR-155 binding site was amplified and cloned into the psiCHECK2 vector (Promega) for wild-type 3′UTR construction. Mutant 3′UTR was established from these wide-type vectors by using Easy mutagenesis system (TransGen Biotech) under the working manuals. All primers were shown in Supplementary Table S1.

H293T cells with 90% confluence were seeded in 12-well plates for co-transfection of 50 nmol/L miRNA oligos and 1 ug constructed psiCHECK2 vectors by Lipofectamine 2000 reagent (Invitrogene). Then luciferase activity assay was conducted by Dual-Luciferase Reporter Assay System (Promega) according to instructions. Firefly luciferase activity was normalized to Renilla luciferase activity, with ratios of firefly luciferase vlaues/renilla luciferase values presented.

### Animal models

Male BALB/c-nude mice, aged 4–5 weeks were purchased from the Experimental Animal Center of Sun Yet-sen University. All experimental protocols were approved by the ethics committee. LV-NC or LV-miR-155 infected cells were harvested by trypsinization and resuspended in PBS, reaching a total number of 3 × 10^6^ per 100 ul. LV-miR-155 cells were subcutaneously injected into the right side, while the control cells were injected into the left side. On day 10, tumors became visible, at a diameter of nearly 2 mm. Afterwards, tumor size was measured every 2 days and calculated by the formula: tumor volume (TV) = length × width^2^ × 0.5 [9]. All nude mice were sacrificed on day 20. Tumor tissues were collected for RNA and protein extraction.

### Statistical analysis

Statistical analyses were performed on SPSS 13.0 software (SPSS Inc.). All quantitative results were presented as the mean ± standard deviation (SD) of three independent experiments. The significance of the differences between two groups, unless for paired comparison which was noted specially, was conducted with a two-tailed Student's *t*-test. The correlations between miR-155 and clinical characteristics were analyzed by Chi-square test. *P*-values < 0.05 were considered to be statistically significant.

## SUPPLEMENTARY FIGURES AND TABLE


