# Supplementary material for: MicroRNA-155 promotes bladder cancer growth by repressing the tumor suppressor DMTF1
Source: Oncotarget. 2015 Apr 18;6(18):16043–58. doi: 10.18632/oncotarget.3755 (PMC4599255; doi:10.18632/oncotarget.3755)
Supplement: Supplementary file 1 [file oncotarget-06-16043-s001.pdf]

SUPPLEMENTARY FIGURES AND TABLE

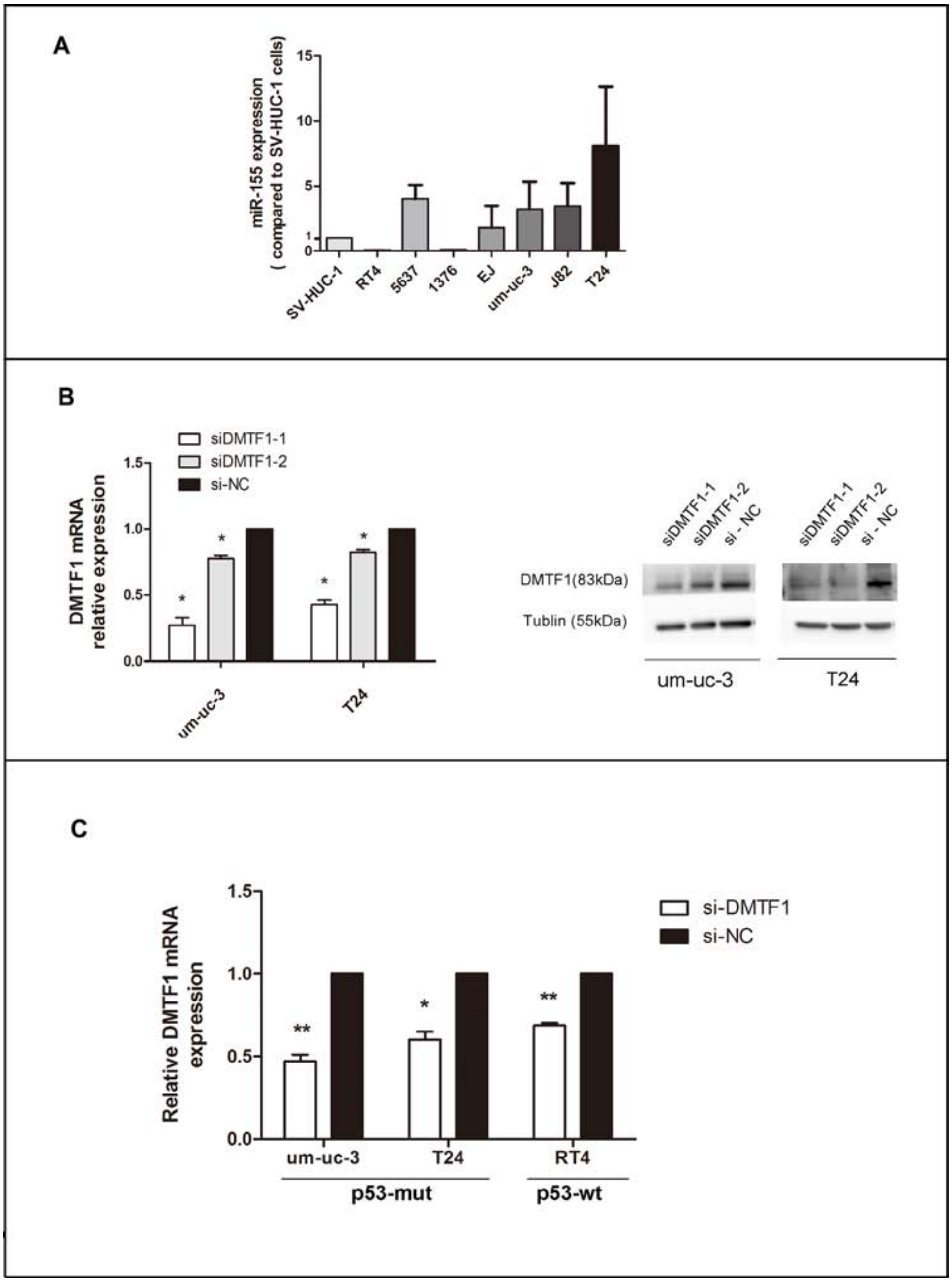

Supplementary Figure S1: A. MiR-155 expression comparison in the 7 bladder cell lines. SV-HUC-1 was used as control. **B.** Western blotting (48 h after transfection) and qRT-PCR (24 h after transfection) validated the DMTF1 RNAi efficiency in T24 and um-uc3 cells; Tubulin was used as protein control and GAPDH was chosen for mRNA internal control, respectively. **C.** DMTF1 RNAi efficiency was confirmed through qPCR in um-uc-3, T24 and RT4, GAPDH was used as control. All results were expressed as the means ± SD; $n = 3$. (*$P < 0.05$; **$P < 0.01$).

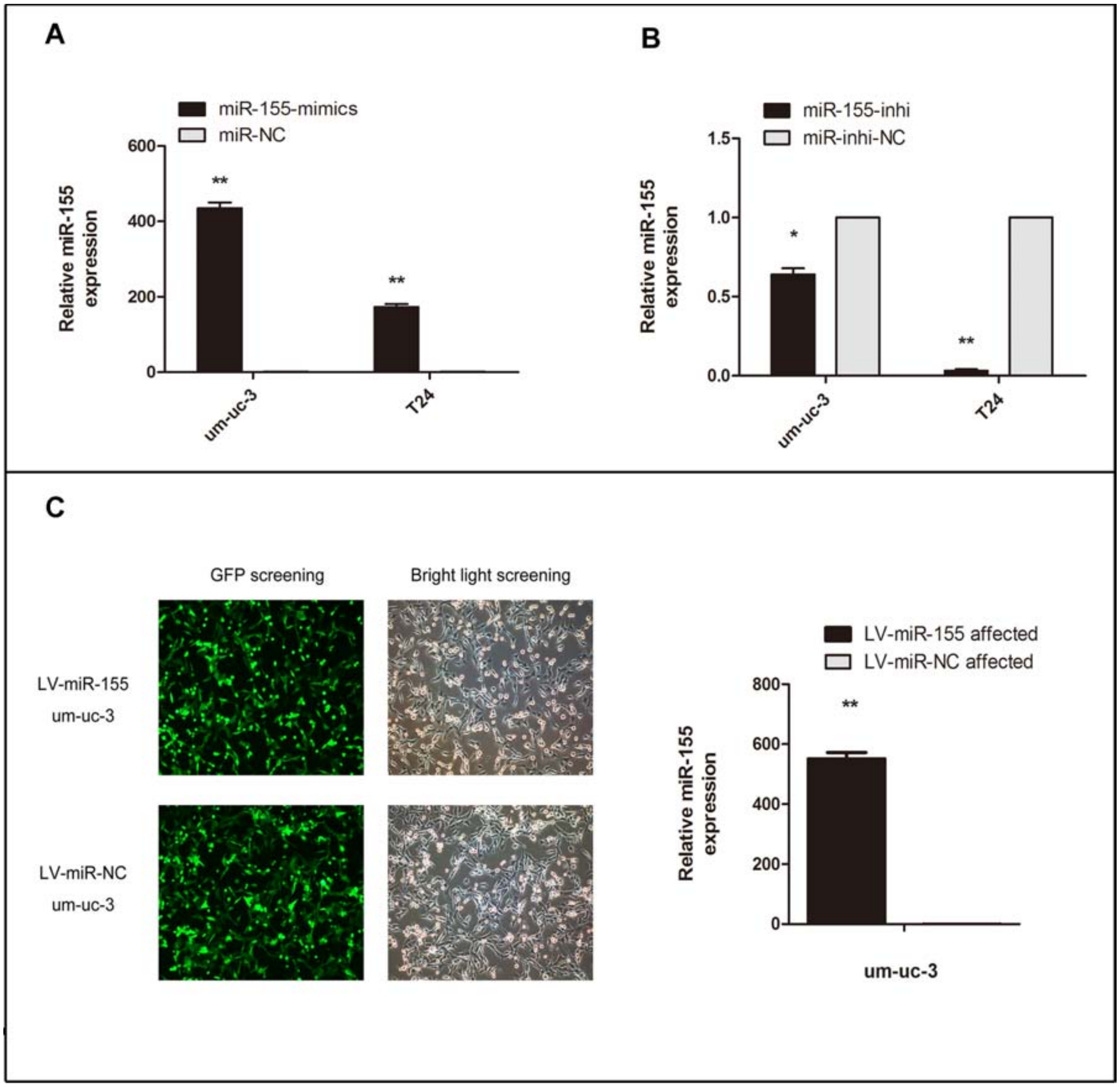

Supplementary Figure S2: A-B. After 24 hours, Transfection efficiencies of both miR-155 mimics and miR-155 inhibitor were validated through qRT-PCR. **C.** 48 hours after infection of lentiviral-miR-155 or lentiviral-miR-NC, each with GFP construct carried, um-uc-3 cells were screened under a fluorescent microscope. qRT-PCR was performed to further confirm miR-155 expression. Data were shown as mean ± SD; $n = 3$, fluorescent and bright light images were representatives of the whole screening. (*$P < 0.05$; **$P < 0.01$).

Supplementary Table S1. Oligonucleotide sequences

Gene Name	Sequence (5′ to 3′)	
qPCR primers		
Has-miR-155	F	TAATGCTAATCGTGATAGGGGT
U 6	F	ACGCAAATTCGTGAAGCGTT
DMTF1	F	GTCTGAACCGGCCTTTGTTTG
DMTF1	R	GCCCAGTCATTGCCATGCT
Arf	F	GATCCAGGTGGGTAGAAGGTC
Arf	R	CCCCTGCAAACTTCGTCCT
p53	F	CAGCACATGACGGAGGTTGT
p53	R	TCATCCAAATACTCCACACGC
p21	F	TGTCCGTCAGAACCCATGC
p21	R	AAAGTCGAAGTTCCATCGCTC
GAPDH	F	GCACCGTCAAGGCTGAGAAC
GAPDH	R	TGGTGAAGACGCCAGTGGA
Clone primers		
DMTF1-WT	F	ATCGCTCGAGAATAATTCTTAGAAATAGGCAGTTC
DMTF1-WT	R	ATCGGCGGCCGCTTGCATCTTAAGACAAATATTCTTT
DMTF1-Mut	F:	TAGGCAGGGTAAACAGGAAACCTTAATAAGTTAAAATTCAC
DMTF1-Mut	R	ATTAAGGTTTCCTGTTTACCCTGCCTAAATCAGCTCCCACTGCCAGCAAT
DMTF1-ORF	F	ATGCCTCGAGGCCACC ATGACTGCAACCACAGAAGTAG
DMTF1-ORF	R	ATGCGAATTCCTAATGACAGTTTACCAAATCTTCGAC
Transfection oligos		
miR-155-mimics	sense	UUAAUGCUAAUCGUGAUAGGGGU
miR-155-mimics	antisense	CCCUAUCACGAUUAGCAUUAAUU
miR-NC	sense	UUCUCCGAACGUGUCACGUTT
miR-NC	antisense	ACGUGACACGUUCGGAGAATT
miR-155-inhibitor		ACCCCUAUCACGAUUAGCAUUAA
miR-inhibitor NC		CAGUACUUUUGUGUAGUACAA
LV-miR-155(mim)		TTAATGCTAATCGTGATAGGGGT
LV-NC		TTCTCCGAACGTGTCACGTTTC
siRNAs		
Si-DMTF1-1	sense	GGAGUUCCAAACAGUAAUATT
Si-DMTF1-1	antisense	UAUUACUGUUUGGAACUCCTT
Si-DMTF1-2	sense	GGAGCCAUCAUUUAAUGAUTT
Si-DMTF1-2	antisense	AUCAUUAAAUGAUGGCUCCTT
Si-Arf	sense	CCCAACGCACCGAAUAGUUTT

(*Continued*)

Gene Name	Sequence (5′ to 3′)	
siRNAs		
Si-Arf	antisense	AACUAUUCGGUGCGUUGGGTT
Si-NC	sense	UUCUCCGAACGUGUCACGUTT
Si-NC	antisense	CCCUAUCACGAUUAGCAUUAAUU

[1]F = Forward primer; R = Reverse primer; WT = wild type 3′UTR; Mut = mutant 3′UTR
NC = negative control; LV = lentivirus.
